# Perception of journal club seminars by medical doctoral students: results from five years of evaluation

**DOI:** 10.3205/zma001525

**Published:** 2022-02-15

**Authors:** Mara Taverna, Julian Nicolaus Bucher, Maximilian Weniger, Roswitha Gropp, Serene M. L. Lee, Barbara Mayer, Jens Werner, Alexandr V. Bazhin

**Affiliations:** 1 Ludwig-Maximilians-University Munich, Department of General, Visceral, and Transplant Surgery, Munich, Germany

**Keywords:** journal club, research paper, critical thinking, post-graduate education

## Abstract

**Objectives: **A journal club is one of the well-established and popular methods of post-graduate education. In this work, we were interested to understand how the participants perceive journal club as a whole and how they evaluate their personal process of acquiring new scientific knowledge and development of soft-skills as an indispensable prerequisite of the lifelong learning.

**Project description:** This study is a survey analysis examining perception of journal club sessions by post-graduate medical students. A checklist for journal club preparation as well as a questionnaire for evaluation of the journal club session by participants has been developed to determine if the journal club had met its aims. Data were collected by summing up all answers to each question of the questionnaire for each session. Qualitative data from a five-year evaluation period were compiled and analyzed.

**Results:** The journal club checklist served as a guideline for the preparation of a journal club session as well as an evaluation questionnaire containing 24 items. Our work presents evidence that journal club seminars are well perceived by participants. Furthermore, a high percentage of participants deemed the working atmosphere to be constructive and found it worthwhile to participate in the sessions. The topics of the presentations have been positively evaluated, however only a minority of participants found that the topics of the journal club was related to their own specific research topic. Concerning the distribution of the journal article, we could show that distributing the paper one week before the journal club event provided sufficient time for preparation. Our evaluation revealed that two-thirds of the participants found discussions during journal club sessions rich and productive. The motivation to think more critically increased during journal club sessions. From our work, it is evident that the participants perceived the speakers´ soft-skills to have improved with the practice. Finally, we show a clear trend of improved perception of the value of journal club sessions from beginning to the end of the evaluation time.

**Conclusion: **Based on the analyzed evaluations, we can conclude that journal club events are highly valued by participants and could be a good option for the development of certain soft-skills.

## Zusammenfassung

**Zielsetzung: **Journal Clubs sind eine etablierte und weit verbreitete Methode der Postgraduiertenausbildung. Das Ziel dieser Arbeit war es zu untersuchen, wie die Teilnehmenden Journal Clubs insgesamt wahrnehmen und wie sie ihren persönlichen Prozess der Aneignung neuen wissenschaftlichen Wissens sowie die Entwicklung ihrer Soft Skills als unverzichtbare Voraussetzung für lebenslanges Lernen bewerten.

**Projektbeschreibung: **Bei dieser Studie handelt es sich um eine Befragungsauswertung zur Untersuchung der Wahrnehmung von Journal-Club-Seminaren durch medizinische Promotionsstudierende. Dazu wurden eine Checkliste für die Vorbereitung von Journal Clubs sowie ein Fragebogen für die Evaluation der Journal-Club-Sitzung durch die Teilnehmenden erarbeitet, um zu ermitteln, ob die Ziele des Journal Clubs erreicht wurden. Die Daten wurden durch Zusammenfassung aller Antworten auf jede Frage des Fragebogens für jede Sitzung erhoben. Qualitative Daten aus einem fünfjährigen Evaluationszeitraum wurden zusammengeführt und ausgewertet.

**Ergebnisse: **Die Journal-Club-Checkliste diente bei der Vorbereitung der Journal-Club-Sitzungen sowie des Evaluationsfragebogens mit 24 Items als Leitfaden. Diese Arbeit liefert Belege dafür, dass Journal-Club-Seminare von den Teilnehmenden positiv wahrgenommen werden. Darüber hinaus empfand ein hoher Prozentsatz der Teilnehmenden die Arbeitsatmosphäre als konstruktiv und die Teilnahme an den Sitzungen als wertvoll. Die Themen der Präsentationen wurden positiv bewertet, auch wenn nur wenige Teilnehmende angaben, dass die Themen des Journal Club Bezug zu ihrem eigenen spezifischen Forschungsthema hatten. Es konnte gezeigt werden, dass eine Verteilung des Zeitschriftenartikels eine Woche vor der Journal-Club-Sitzung ausreichend Zeit zur Vorbereitung bot. Die Auswertung ergab, dass zwei Drittel der Teilnehmenden die Diskussionen während der Sitzungen bereichernd und fruchtbar fanden. Die Motivation für kritischeres Denken stieg während der Journal-Club-Sitzungen. Gestützt auf diese Arbeit ist es evident, dass sich die Soft Skills der Vortragenden in der Wahrnehmung der Teilnehmenden mit zunehmender Übung verbesserten. Schließlich konnte ein eindeutiger positiver Trend bei der Wahrnehmung des Nutzens von Journal-Club-Sitzungen vom Beginn bis zum Ende des Evaluationszeitraums nachgewiesen werden.

**Schlussfolgerung: **Auf Grundlage der ausgewerteten Evaluationen kann geschlussfolgert werden, dass Journal-Club-Seminare von den Teilnehmenden sehr geschätzt werden und eine sinnvolle Möglichkeit für die Entwicklung bestimmter Soft Skills sind.

## Introduction

The authors of this paper define the term “journal club” as a regular meeting of individuals to discuss a research paper published in a scientific journal. A journal club (JC) is one of the well-established and popular methods of graduate and post-graduate education with about two hundred years of history [1]. Originally, its purpose was to achieve a better dissemination of scientific information in a low-cost manner [2]. With time, JCs became accepted as an educational intervention [3] with attendance becoming part of requirements to be fulfilled to obtain a doctoral degree. In some universities, a JC is anchored in curricula of their structured doctoral programs [4]. A JC is primarily a face-to-face interaction. However, with the immense influence of internet these days, there is a trend to carry out JCs in an on-line format or even to use social media [1], [5]. With the advent of the COVID-19 pandemic in 2020, there has been a huge impetus to this trend due to the high importance of physical distancing [6], [7], [8]. 

Many aims can be achieved by participants during JC sessions. The first and oldest aim is the spreading of scientific information and knowledge transfer, so that the participants are keeping up to date with the literature [9], [10]. The second aim is to help develop the ability to critically appraise the literature [11] as well as to develop critical thinking or analytical skills in general [12], [13]. In the case of MD students, it is believed that JC sessions may improve their knowledge of evidence-based medicine and help them translate this process to clinical practice [14], [15]. Moreover, generic (interdisciplinary) skills or so-called soft-skills such as communication, leadership, teamwork, moderation, discussion, time-management, critical thinking and debating can also be improved by JC [13], [16]. Depending on the purpose and criteria, the evaluation of the success of a JC can be carried out in different ways. The number of participants which normally ranges from 12 to 135 participants [17], would be for example the easiest way to evaluate a JC success [4]. A complicated approach would be to evaluate the development of critical appraisal of the literature or assess learning outcome pre- and post-test or in a randomized trial with control and experimental groups [17]. 

We can categorize research to date on JC as an education tool into three major groups:

1. JC as a cognitive tool to improve critical thinking [12], [18], [19], [20], [21] and evidence-based skills [22], [23], [24], [25], [26], [27];
2. strategies to improve the effectiveness of JC [28], [29], [30], [31], [32]; and
3. new approaches to carry out JC [8], [33], [34], [35], [36], [37], [38].

When it comes to the role of JC in the development of soft-skills, research on this topic is very scant. In our work, the main aim was to understand how the participants perceived JC as a whole and how they evaluated their personal progress in acquiring new scientific knowledge and soft-skills.

## Project description

### The study and participants 

This study is based on a survey analysis that explored the perception of JC by post-graduate medical students who performed their MD thesis at the Department of General, Visceral and Transplant Surgery of the Ludwig-Maximilians-University (LMU) Munich. The JC evaluation was started in the winter semester of 2014 and ended in the summer semester of 2019. Forty doctoral students attended JC sessions on a regular basis and all attendees filled out an evaluation form after each session. JC sessions were carried out every second week of the academic year, starting from October and ending in July of the following year. Participation in the JC sessions was semi-obligatory, that means the students have to attend 50% of all sessions and have to be a speaker at least once during their doctorate. Therefore, the group was open and the constellation of participants was not the same during the whole time under observation. In addition, some but not all of the students participated in JC sessions up to 3 years from the beginning of their doctoral degree. According to our local ethics commission from the Medical Faculty of LMU Munich, this study did not need any formal ethical approval. All data were completely anonymized. The agreement (signed consent form) for usage of the evaluation results for scientific purposes has been collected from the participants. 

#### Preparation and carrying out of JC sessions 

**Figure 1:**
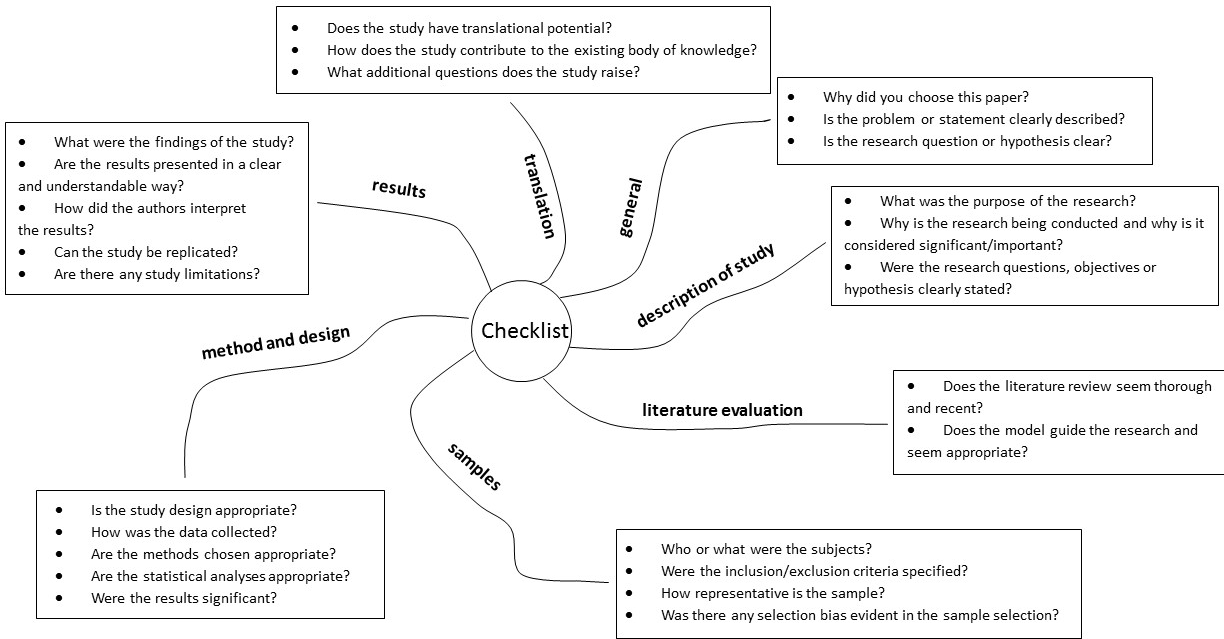
Checklist for journal club preparation

The first JC session of each academic year was presented by an experienced scientist (one of the authors; MT, RG, SL, BM or AVB). They not only provided an example of how the students should perform their JC session, but also presented and discussed the aims of a JC and the hallmarks of a successful JC for both the presenter and the attendees. Our JC format is a didactic mixture of the classic and flipped classroom forms [39]. Speakers chose papers for presentation (after consulting their supervisor, if necessary). While there were no strict rules for the choice of papers, the students have to follow a provided checklist (see figure 1 (Fig. 1)) for preparation of a JC session. The paper to be discussed had to be distributed to the other participants at least one week before the JC session to allow participants sufficient time for critical reading. The presentation format was left open, but usage of PowerPoint for presentation was not recommended in order to avoid projecting the feel of a lecture. After presentation of the paper (15-20 min), a discussion was initiated and moderated by the presenter (15-20 min). An experienced scientist with additional didactic background (MT or AVB) attended every session and helped the speaker, if required, to moderate the discussion (learning by example). After each JC session, the participants had to fill up a questionnaire anonymously. The data from the questionnaires were analyzed after each session by MT. The last JC session, moderated by the corresponding author (AVB) has been devoted to the discussion of the anonymous evaluation results to identify possible problems and make changes, where appropriate, for the next academic year.

#### Preparation of a checklist 

A checklist with guidelines for preparing the JC was developed during a workshop attended by the authors of the paper and students in autumn 2014. The corresponding author (AVB), who has a strong background in adult learning theory and high school didactics, moderated the workshop. After AVB’s presentation of current literature on the efficacy of JC mentioned above [2], [3], [4], [9], [10], [11], [12], [14], [15], [16], [17], [18], [19], [22], [28], [29], [40] and the desired learning objectives, the workshop participants worked in small groups to brainstorm and elaborate on the content of the checklist. The JC checklist (see figure 1 (Fig. 1)) served as a guideline for the preparation of a journal club session by the designated speaker. The checklist was presented at the first introductory JC session each year and discussed thoroughly with participants.

#### Preparation of a questionnaire 

A questionnaire for JC evaluation by participants was developed by the authors of the paper (SL, BM and AVB) in a topic-centered workshop based on literature cited in the previous paragraph. The results of the workshop have been judged to be conforming to the quality management standards of our ISO 9001:2015-certified Department. The developed evaluation questionnaire (see table 1 (Tab. 1)) consists of six groups of questions (levels of evaluation, see table 1 (Tab. 1)) which correspond to sub-goals of the project, evaluating

1. the JC as a whole,
2. topic of the presentation,
3. process of a JC session,
4. participants’ perception of their learning process,
5. assessment of speaker’s soft-skills during the presentation,
6. perception of participation of colleagues in the JC session.

Each group of questions in the questionnaire comprised of three to seven questions. In total, 24 items have been included (see table 1 (Tab. 1)) ranging from 1 (“does not apply”) to 5 (“true”) on the Likert scale. Reliability of the internal consistency of the questionnaire has been assessed by Cronbach’s correlation analysis. Next, the questionnaire was assessed for face (subjective assessments of relevance, reasonability and unambiguity) and content (an exhaustive literature review to extract the related items) validity before circulation [41]. 

A questionnaire was distributed to each student at every JC session. All answers to each question of the questionnaire were summed up for each session. For each question, the number of participants who checked a positive, negative or neutral category from the Likert scale was tallied up separately. Answers of “does not apply” and “does rather not apply” were merged, as well as answers of “rather apply” and “true”. The merging of the two “positive” categories and the two “negative” categories is usually done to get a cleaner picture of the results from a Likert scale without losing information. Graphics and analyses were done using GraphPad Prism 5 (GraphPad Software, Inc.).

## Results

### General evaluation of questionnaire development, JC sessions and participants

The internal consistency of the questionnaire is high as indicated by a Cronbach alpha coefficient of 0.97. The number of JC sessions and participants each year is summarized in table 2 (Tab. 2). It should be noted that the evaluation of the JC sessions in the first year was only done during the summer semester and not during both summer and winter semesters as for the other years. Therefore, the number of evaluated JC sessions in the first year of observation was lower than in the following years. However, the number of participants in the first year was higher than in the next ones and was relative stable in the following years (see table 2 (Tab. 2)).

#### General evaluation of the JC sessions 

**Figure 2:**
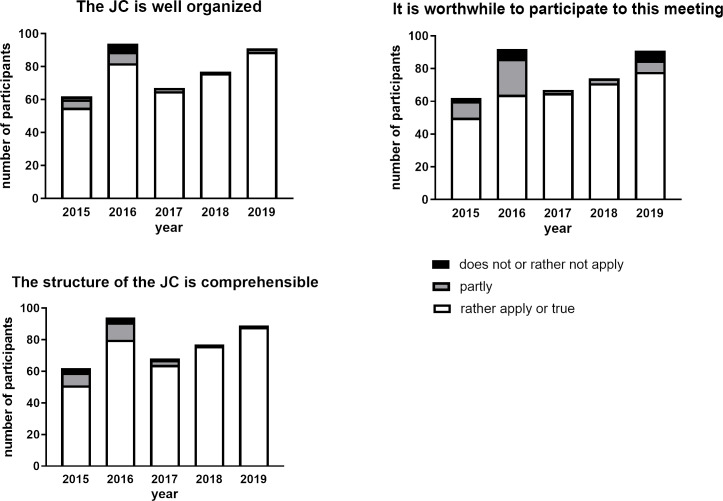
Evaluation results when assessing the journal club as a whole. Number of participants on the Y-axis means the amount of students attended and evaluated several JC sessions.

First, the participants had to evaluate the JC session as a whole (i.e. organization, comprehensibility and a general judgment about the value of this event). In general, the JC as a whole has been perceived positively (“rather apply” or “fully applies” was checked by more as 70% of all participants). It should be stressed that a tendency to more favorable perception of the JC started from 2017 and the perception was increasingly favorable and then remained stable during the next years of evaluation (see figure 2 (Fig. 2)). 

The comprehensibility of the JC structure should be reflective of the JC checklist mentioned above. At the beginning of the observation period (years 2015-2016), the percentage of participants who evaluated the JC structure as comprehensible was about 80% and increased at least to 95% in the following years (see figure 2 (Fig. 2)).

Also at the beginning of the evaluation, the percentage of participants who answered with “rather or fully apply” to the statement “it is worthwhile to participate in this meeting” was in the range of 70-80%. However, in the next years this percentage increased and reached maximum of 95% of the participants in the year 2016 (see figure 2 (Fig. 2)). 

#### Evaluation of topic of the presentation

**Figure 3:**
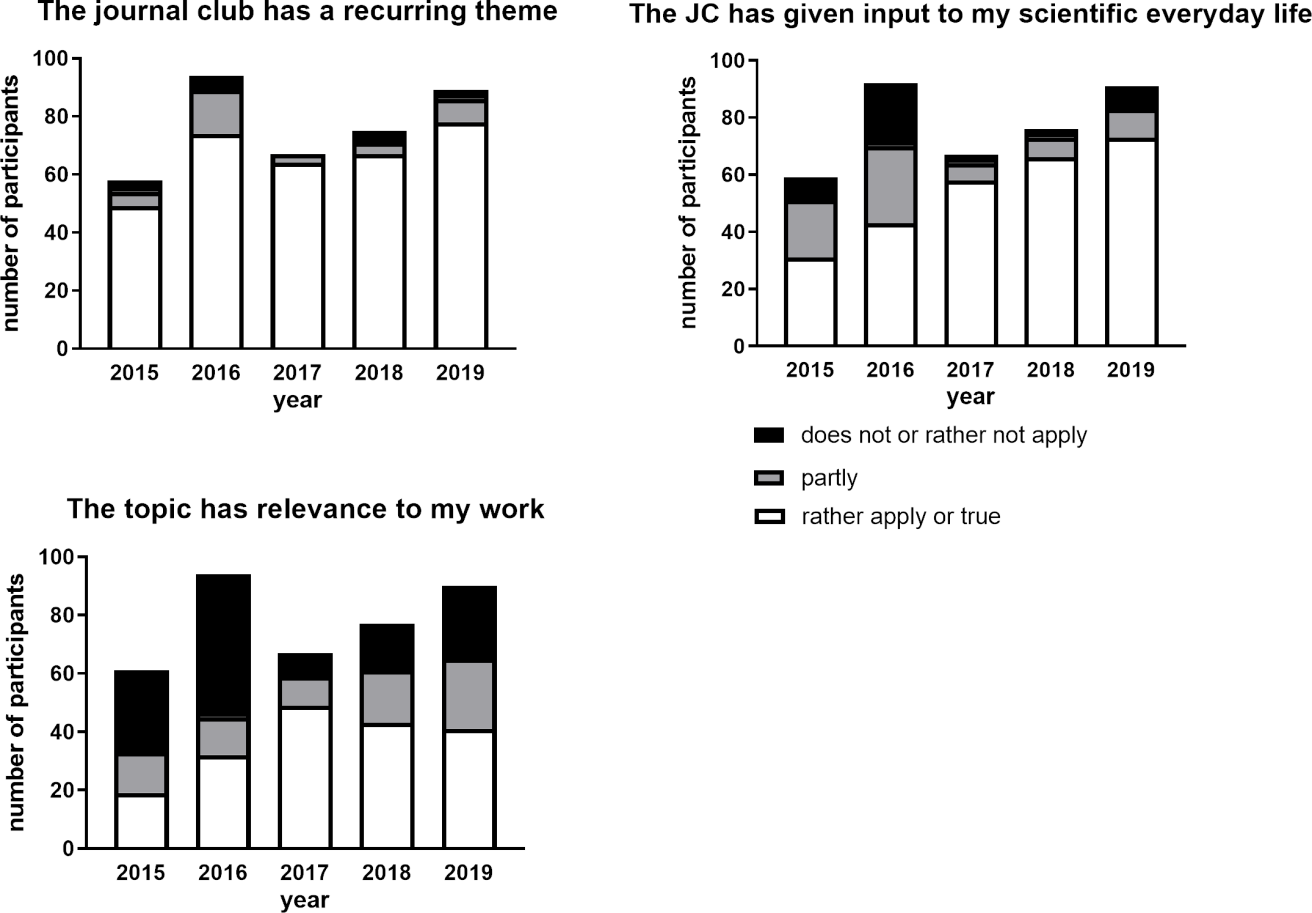
Evaluation results when appraising the topic of journal club sessions. Number of participants on the Y-axis means the amount of students attended and evaluated several JC sessions.

A good choice of the paper for discussion is a prerequisite of a successful JC session [13]. The next group of questions served to evaluate the chosen topic to be discussed during the JC. More than 75% of the participants recognized that the JC sessions had a recurring theme (see figure 3 (Fig. 3)). However, the relevance of this topic to their own research was low, especially in 2015 to 2016 (see figure 3 (Fig. 3)), which could be partially explained by heterogeneity in the scientific projects of participants. At the beginning of the JC evaluation (2015-2016), only about 50% of participants agreed with the statement that the JC has given input to their scientific everyday life. However, from the third year of evaluation, more participants (about 80% of participants) agreed with the statement above (see figure 3 (Fig. 3)). 

#### Evaluation of a JC session as a process

##### Timely distribution of the paper

**Figure 4:**
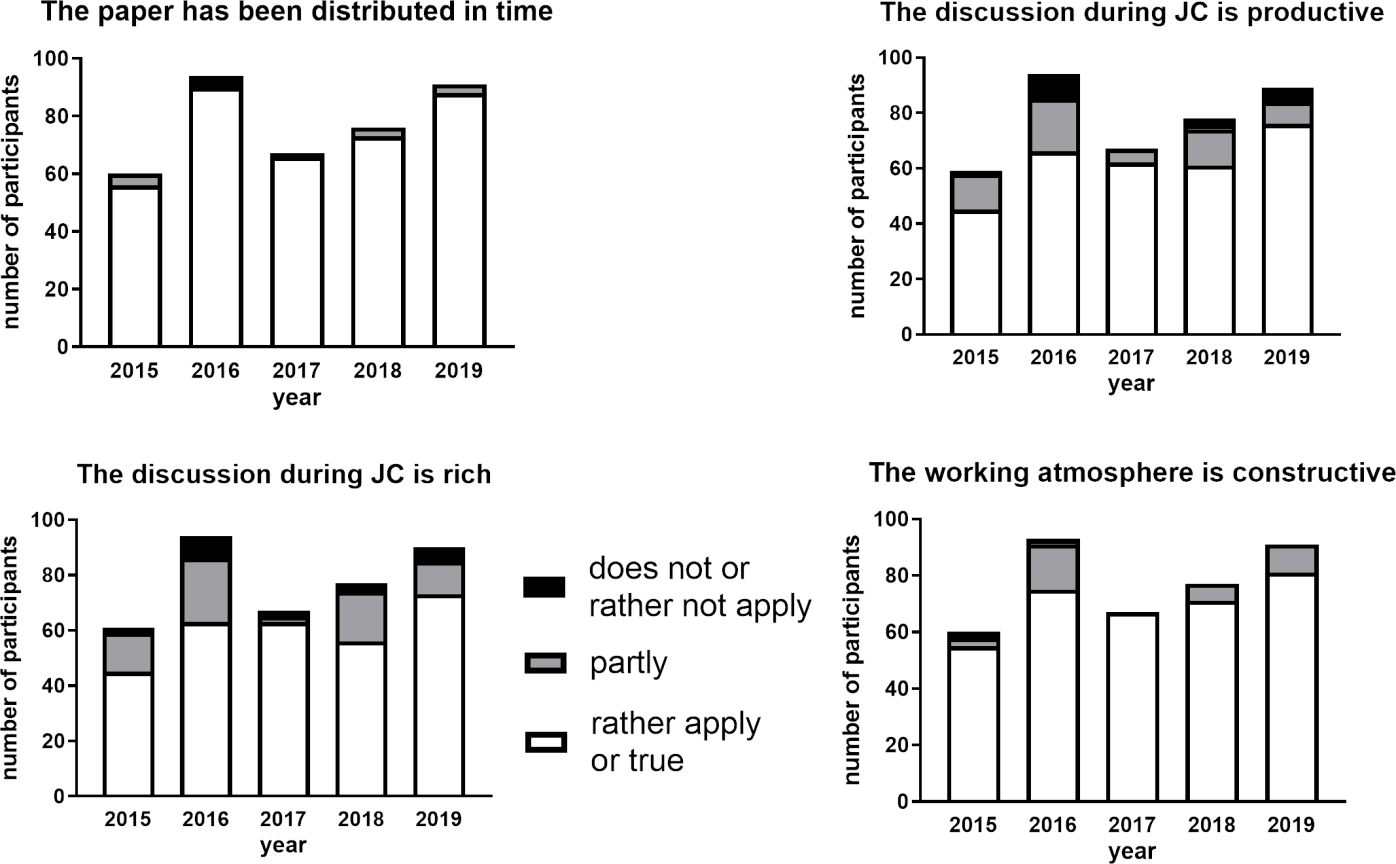
Evaluation results when considering the process of journal club sessions. Number of participants on the Y-axis means the amount of students attended and evaluated several JC sessions.

An important requirement for a successful JC session is the timeline behind the distribution of the paper [17]. When a paper is distributed in time, participants have sufficient time to read the paper conscientiously, understand the paper to the best of their ability and pinpoint sections where they feel require further discussion to meet their learning objectives. The evaluation revealed that in most cases the papers have been distributed in time (see figure 4 (Fig. 4)). To ensure that the process of the JC is successful, it is important that the working atmosphere is constructive [12]. Indeed, about 100% of the participants in the JC from 2017 found the working atmosphere constructive. In contrast, only 80% of the participants answered with “rather or fully applies” in 2016 (see figure 4 (Fig. 4)). 

##### Constructive discussions during JC sessions

Discussion during a session is also an important part of the JC process [17]. Therefore, we asked participants to rate the discussions during the JC to indicate whether or not the discussion was rich. The evaluation results were heterogeneous: about 75 to 80% percent of participants found the discussions sufficient in the years 2015, 2016, 2018 and 2019. However, in the year 2017 more than 90% of the participants found that the discussion during the JC was rich (see figure 4 (Fig. 4)). A rating of abundant discussion does not indicate to us that the discussions were productive. Therefore, ratings on the productivity of the discussions during JC sessions have also been recorded. The evaluation of the responses revealed that the result had the same trend as the responses to the previous question on whether or not the discussions were rich and positive responses were at a maximum in year 2017 (see figure 4 (Fig. 4)). 

#### Evaluation of the learning process

##### Learning objectives reached

**Figure 5:**
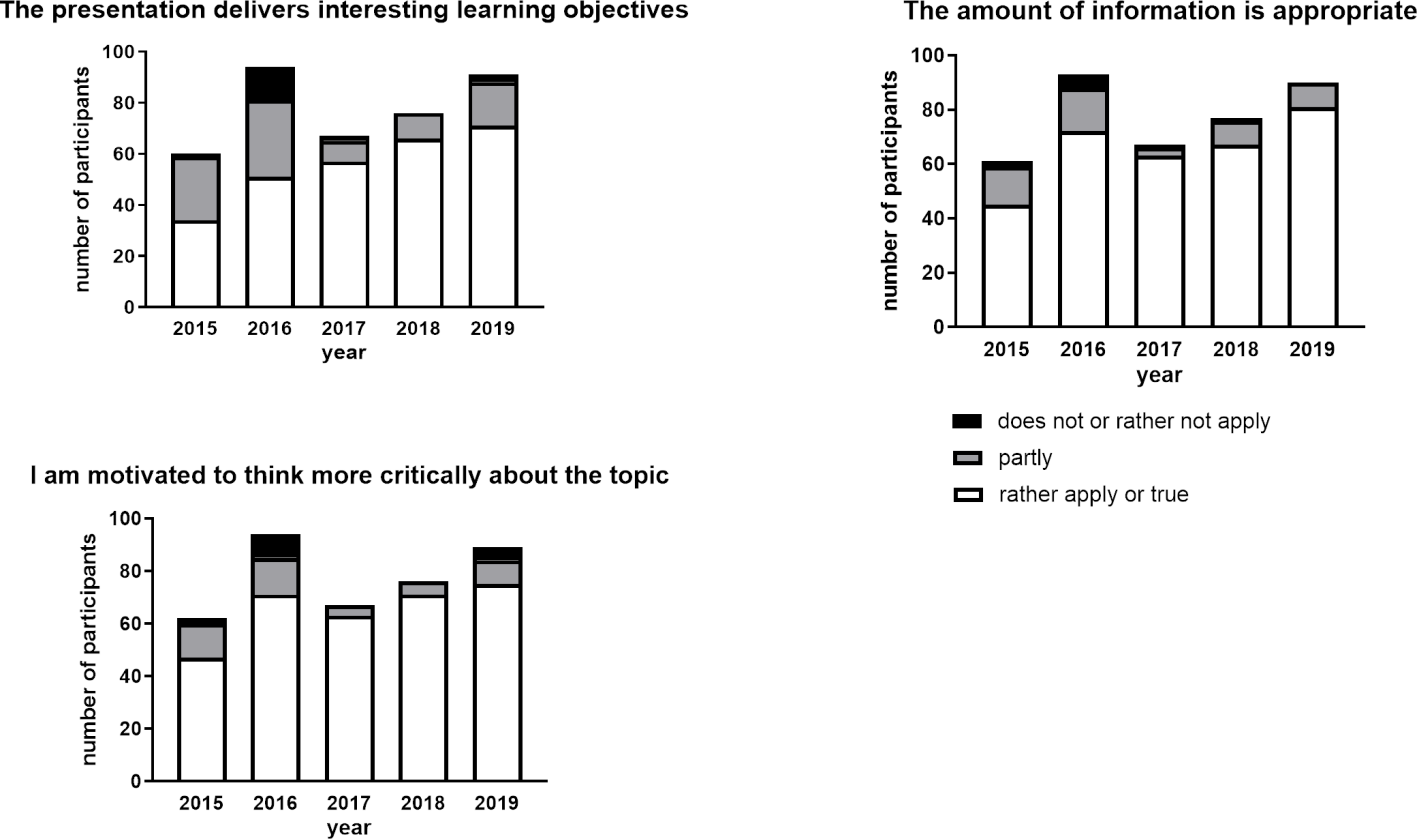
Evaluation results when assessing one’s own learning process. Number of participants on the Y-axis means the amount of students attended and evaluated several JC sessions.

Regarding the learning process, it was important for us to understand how students evaluate their own learning process due to participation in the JC. Since interest seems to be an important factor of learning [40], we asked participants whether the JC presentation can deliver interesting learning objectives. The evaluation revealed that at the beginning of carrying out our JC project in years 2015 and 2015, only about 55% of the participants think that the presentation indeed brings them interesting material for learning (see figure 5 (Fig. 5)). However, from 2017 another tendency has been observed: more than 80% of the participants found that the JC can bring interesting learning objectives. This trend stayed nearly the same throughout the following years (see figure 5 (Fig. 5)). 

##### Motivated to think critically

In this study, we did not analyze the influence of participation in a JC on development of critical thinking of our students. Nevertheless, we aimed to understand, how our participants evaluated their motivation to think more critically about the issue discussed in the JC. In the first two years, the perception that they were motivated to think more critically was evaluated as “rather or fully applies” by around 75% of the participants. From 2017, this percentage was increased until it reached a maximum 95% (see figure 5 (Fig. 5)). 

##### Appropriate amount of information for learning

The amount of supplied information to be learned has an impact on learning process. Therefore, we asked our participants if the amount of information in each JC session was appropriate. In the first years that JC sessions were evaluated, about 75% of participants found that the amount of information shared in the JC was appropriate. This percentage was increased in the next years to a maximum of 95% of the participants in 2017 (see figure 5 (Fig. 5)). 

#### Evaluation of the speaker's soft-skills during the presentation 

**Figure 6:**
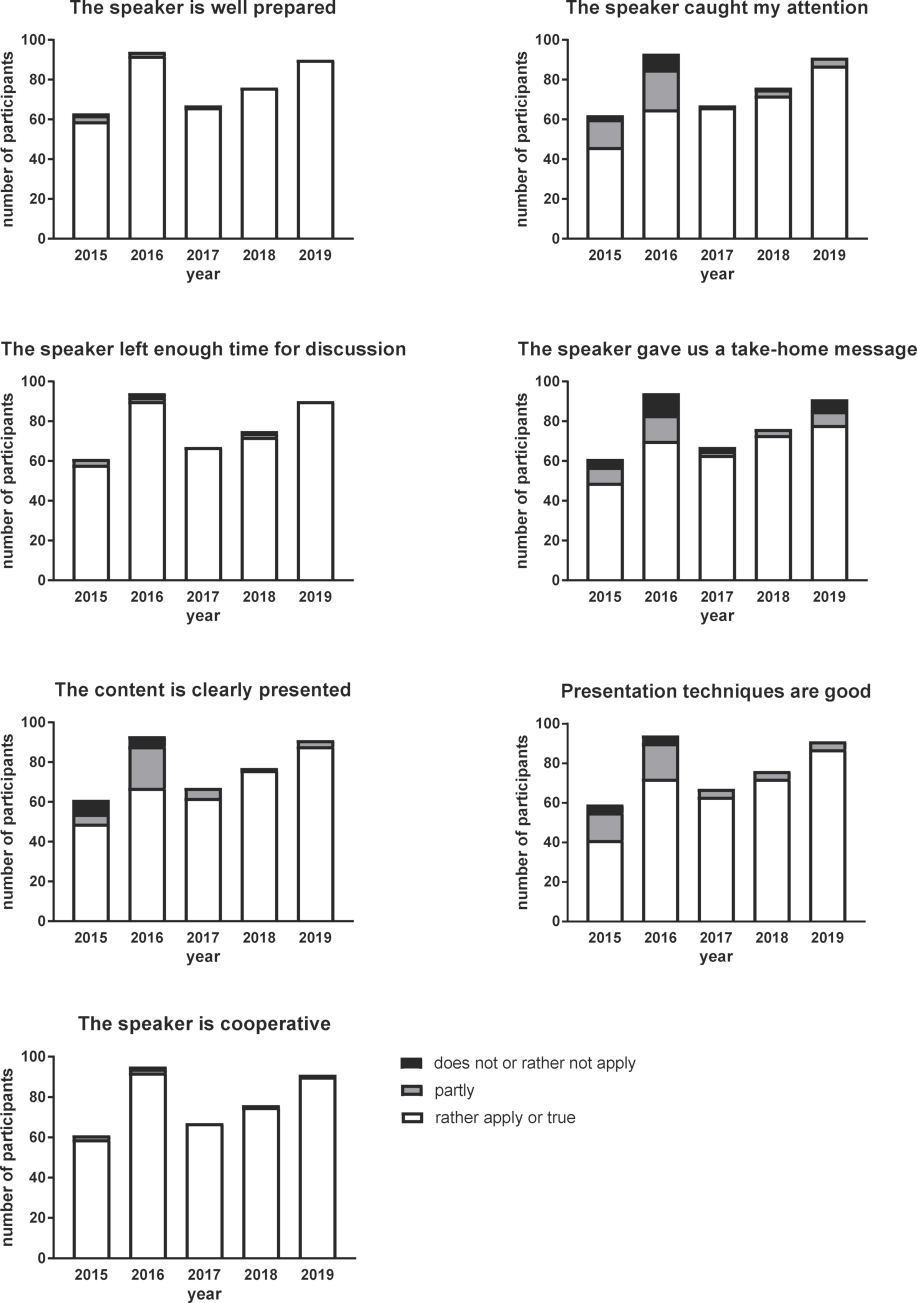
Evaluation results of rating the speakers’ soft-skills. Number of participants on the Y-axis means the amount of students attended and evaluated several JC sessions.

A JC is recognized to be platform for the development of certain soft-skills (presentation, moderation, debate, time-management, leadership etc) [42], which are no doubt important for scientific employability. Therefore, participants have evaluated the speakers’ soft skills after JC sessions. In general, the participants (95% in year 2015 and up to 100% in the last two years of observation) found that the speakers were well prepared (see figure 6 (Fig. 6)). However, in the first years that JC sessions were carried out, only 70 to 75% of the participants stated that the speaker had caught their attention. However, this percentage increased to 95% of the participants in the following years of observation. This result is reflected in the next questions on whether the speaker gave a take-home message and whether the content was clearly presented (see figure 6 (Fig. 6)), underscoring the importance of presentation skills. Presentation techniques were perceived to be good by 70 to 75% of the participants at the beginning of evaluation period. The percentage then continued to increase from 2017 and it stayed stably high in the last years of observation (see figure 6 (Fig. 6)). 

The importance of discussion has been already mentioned above. To leave enough time for discussion is also an important soft-skill related to moderation, time-management and leadership. A minimum of 95% of participants replied to the statement “the speaker left enough time for discussion” with “rather apply or true” through all the years of observation (see figure 6). Cooperation can be recognized to be a trait of leadership that is also important in JC settings [13]. Indeed, nearly all participants found that the speakers were cooperative during JC sessions (see figure 6 (Fig. 6)).

##### Evaluation of the participant's perception of other participants attending the JC sessions

**Figure 7:**
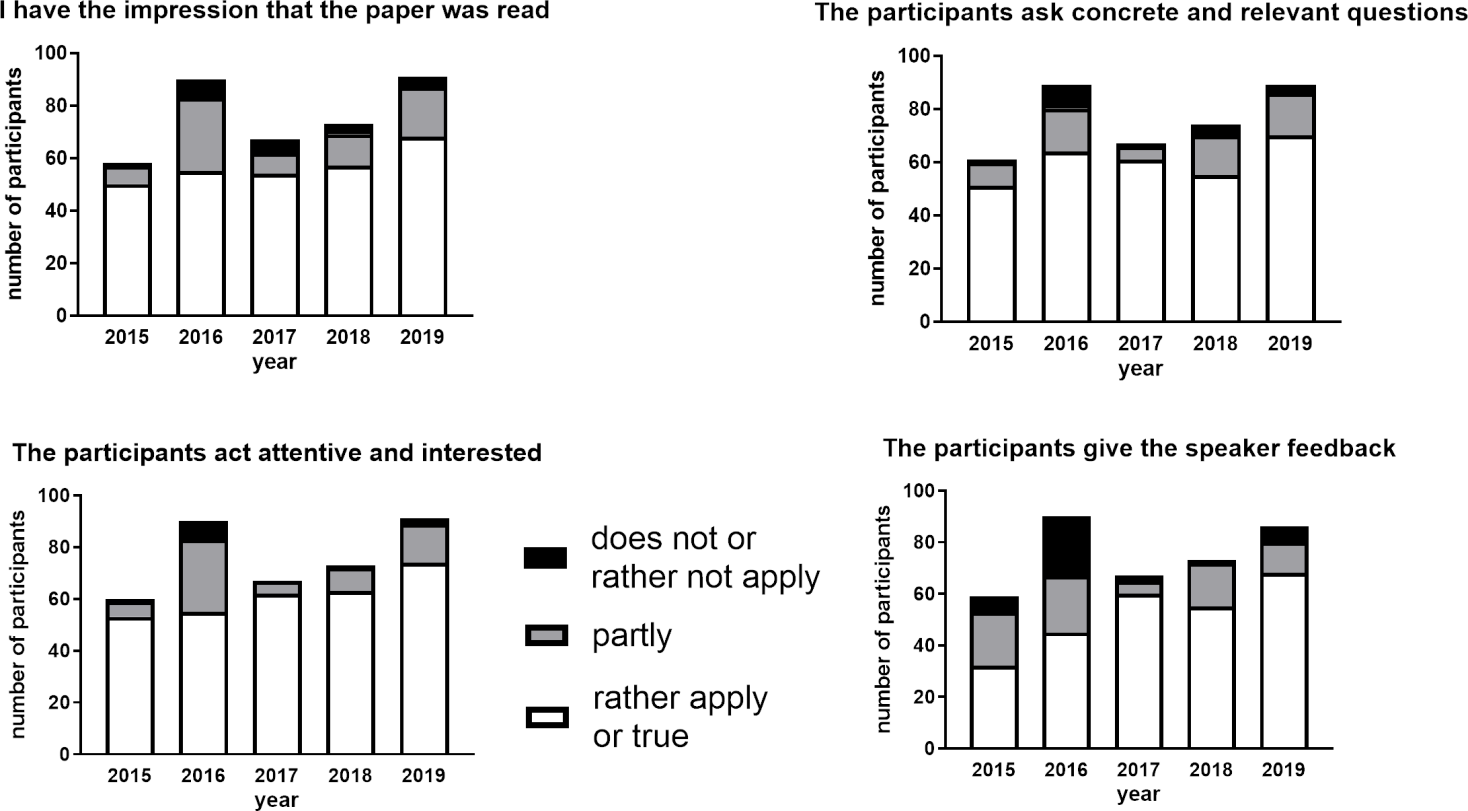
Evaluation results summarizing the participant’s perception of colleagues attending the journal club sessions. Number of participants on the Y-axis means the amount of students attended and evaluated several JC sessions.

Lastly, we were curious to find out how the participants perceive their colleagues attending the JC sessions. In 2016, only about 60% of participants had the feeling that the paper distributed before the JC session had been read. In contrast, 85% of participants in 2015 felt that papers have been read (see figure 7 (Fig. 7)). Practically the same percentage distribution was recorded for the statement “the participants act attentive and interest” and “the participants ask concrete and relevant question” (see figure 7 (Fig. 7)). 

Positive feedback provides extrinsic motivation and builds self-esteem [43]. In the context of a JC, this is a strong motivation to be a speaker and more importantly be a good speaker. Therefore, we monitored whether the participants gave the speakers feedback during his or her JC session. At the beginning of the evaluation period, only about 50% of participants felt that the speaker obtained feedback. From 2017, this situation changed and 75 to 90% of the participants responded that speakers indeed received feedback from their colleagues. 

## Discussion

JC seminars are becoming more and more important not only in the context of spreading scientific information but also in the field of education. As such, this seminar format is increasingly popular for training post-graduate students especially in structured post-graduated curricula. As mentioned before, the number of participants can be a criterion to measure JC effectiveness. However, this measure can only be used when JC seminars are not compulsory to attend. We know from the systematic review of Deenadayalan and colleagues [17] that the number of participants can vary in the range of 12 to 135. In the case of our JC events, where a minimum attendance rate is obligatory, we have a lower number of participants. When attendance is not compulsory, we think that the attendance and number of participants depend on many factors: number of research groups, time-frame of events, topic etc. This point must be considered when planning to include JC seminars in curricula. 

Even though Deenadayalan et al. mentions that evaluation of JC effectiveness should be specifically related to the paper discussed [17], we think that the participants’ positive perception of the JC as a whole as well as the knowledge that they are gaining important skills which are not directly related to the paper (i.e. soft-skills), are also important to increase the motivation of students to attend JC sessions. This is an important point because JC sessions, as in our case, are not always a non-obligatory event. Making attendance a prerequisite for graduation could make JC an unpopular activity. On the contrary, our work presents evidence that such seminars are actually well perceived by participants. This result indicates that the general meaning and value of the JC is well-understood by participants [18]. Furthermore, a high percentage of participants deemed the working atmosphere constructive and mentioned that it was worthwhile to participate in JC sessions. 

Keeping in mind that a JC should ideally contribute constructive inputs to scientific everyday life, choosing an appropriate article is believed to be another important factor of JC effectiveness [12], [13], [17]. Important criteria that can be used to choose an article include whether it represents a recurring topic and whether it is relevant to the work of other participants. If the topic of the presentation has been positively evaluated, this correlates to a higher percentage of participants, finding that the theme of the JC is related to their own specific research topic. In our case, these data were easy to interpret, since the designated speakers were free to choose the paper themselves after a consultation with their supervisors. Therefore, the topic of the presentation was related to their own research topic. However, the research groups in our Department are heterogenous and thereby have different scientific interests. Therefore, according to our expectations, the evaluation results revealed that the scientific topics often had only a low relevance to the individual participant’s scientific topic. However, we recommend that organizers of JC seminars strategically define the criteria for choosing publications for discussion so that all participants will benefit even in the context of scientific heterogeneity of the hosting department. 

The above-mentioned heterogeneity of participants from different working groups raises the question of whether an effective JC can be attained in this circumstance. On one hand, Deenadayalan et al. recommend that the chosen paper should cover the particular interest area of all participants [17] and on the other hand, Lonsdale et al. writes that “Multidisciplinary is not a dirty word” (p. 3) and to “Let your topics be as diverse as your members” (p. 4) [13]. We share the opinion of Lonsdale and colleagues, since the heterogeneity of a (learning) group can promote the learning process evoking different interests and increase motivation to attend.

In fact, Deenadayalan et al did not provide a definitive answer to the question about the importance and necessity to distribute the paper prior the JC session. However, they recommend to provide all participants with enough time for reading [17]. Nevertheless, the amount of time that is considered enough is not defined. Our participants have been instructed to send their paper of choice by e-mail at least one week before the JC session. Nearly all participants have responded that this amount of time was enough for preparation. Therefore, we recommend distributing papers to be discussed a minimum of 7 days before the event. 

Taking into consideration the learning process of participants, there are some investigations dealing with learning outcomes. This research was summarized in the systematic review by Deenadayalan et al. They concluded that some data showed significant improvement of professional skills (i.e. biostatistics) or even of critical appraisal abilities [17]. We wanted to understand how participants perceive their own learning process due to JC. As interest is a motivator of learning [40], we were curious to know whether participants have a feeling that the JC presentation can supply them with interesting learning objectives. Interestingly, at the beginning of the evaluation only half of the participants found that the presentation is of interest in the context of their learning objectives, but from the third year of the evaluation, this percentage of respondents increased. Richness and productivity of discussions could be factors that make a JC successful and contribute to the learning processes. However, to our knowledge, there is no data about perception of discussion during a JC session in the literature so far. Our evaluation revealed that in our case about two-thirds of the participants found discussion during JC sessions rich and productive. The impact of discussions during JC sessions on its effectiveness should be investigated further in the future, especially in the context of learning processes.

As mentioned before, we did not aim to analyze the influence of JC sessions on the development of critical thinking in our participants. Instead, we wanted to survey their self-assessment of whether or not JC participation can motivate them to think more critically about the literature read. While only half of all participants rated themselves well at the beginning of the evaluation period, the percentage rating themselves as being more motivated to think more critically increased as time progressed. This perception of self-motivation to think more critically is in line with research data showing that JC indeed improves critical appraisal of literature by participants [17] as part of a whole critical thinking process. 

Another interesting point to discuss is how much information could be delivered and processed by participants during a JC session. If we overload participants with a huge amount of information, they would not be able to deal optimally with the complexity of the matter, especially as participants are not established scientists yet. From the other side, the amount of information must be enough to motivate their learning. The results of the evaluation show that in our case, most of the participants think that they were getting enough information to be challenged but not too much such that they are overwhelmed. This topic about how to convey an appropriate amount of information during a JC session should be also investigated in future. 

Soft-skills are indispensable prerequisites for lifelong learning and as such are important for future employability [44] and they can be promoted through a JC [13], [16]. From our work, it can be seen that the soft-skills of speakers in general as perceived by the participants had improved with practice. Therefore, we can agree with Fowler, Lonsdale and their colleagues that soft-skills can also be developed by participation in a JC as a speaker. However, pre- and post-survey analyses are needed to support this statement. 

The attitude of colleagues participating in JC sessions can be motivating or even de-motivating for participants. Therefore, an interesting part of our questionnaire was an evaluation of the participants' perception of their colleagues in the JC session. By analyzing the evaluation form, we learned that there is a feeling that the papers distributed for preparation were not always read. This result is in contrast to the evidence that one week for preparation for JC is enough. Therefore, more research is needed to understand this contradiction. Nevertheless, this fact did not impair the interest of the students to take part in the event. Finally, the evaluation showed that feedback was still desired. 

### Implications for implementation of a journal club

An important implication from our work is the importance of regularly evaluating JC. However, the exact data collected for evaluation should be defined by the organizers of the JC based on their desired purposes and learning outcomes. Based on our requirements and aims formulated as mentioned previously, we created a questionnaire that could serve as a template for other institutions. Finally, carrying out goal-setting workshops before the official start of JC and ensuring that the JC is supervised by someone experienced, who holds teaching qualifications for university education, is highly recommended. 

#### Strengths and limitations 

This study has certain limitations: Firstly, it was conducted in a one-center setting making generalization of the results obtained challenging. Secondly, the value of the Cronbach alpha coefficient was more than 0.95 which might point to a certain amount of redundancy between questions. Thirdly, we had new students matriculating and final year students graduating in the examined period, therefore we cannot conclusively say that the effects seen were due to an improvement of the participants’ skills during the time. We also cannot exclude that this effect could be attributed to the extrinsic motivations of the participants. Finally, the questionnaire was designed to be completely anonymous; therefore, the demographics were neither collected nor analyzed, which might also have an impact on the generalizability of the results. The important strengths of this study are the high number of JC sessions evaluated as well as the longitudinal observation. Due to these strengths, we saw a clear trend of improved perception of JC sessions from the beginning of the evaluations in practically all of the items in the questionnaire.

## Conclusions

Based on the analyzed evaluations, we can conclude that journal club events are highly valued by participants and could be a good option for the development of certain soft-skills. 

## Abbreviations

- ISO = International Organization for Standardization
- JC = Journal club
- LMU = Ludwig-Maximilians-University
- MD = medical doctor

## Acknowledgements

The authors would like to thank Ms. Dana Dacian for her excellent administrative assistance in the organization of journal club sessions. We acknowledge Prof. Dr. Matthias Siebeck for his valuable advice regarding data presentation. 

## Competing interests

The authors declare that they have no competing interests.

## Figures and Tables

**Table 1:**
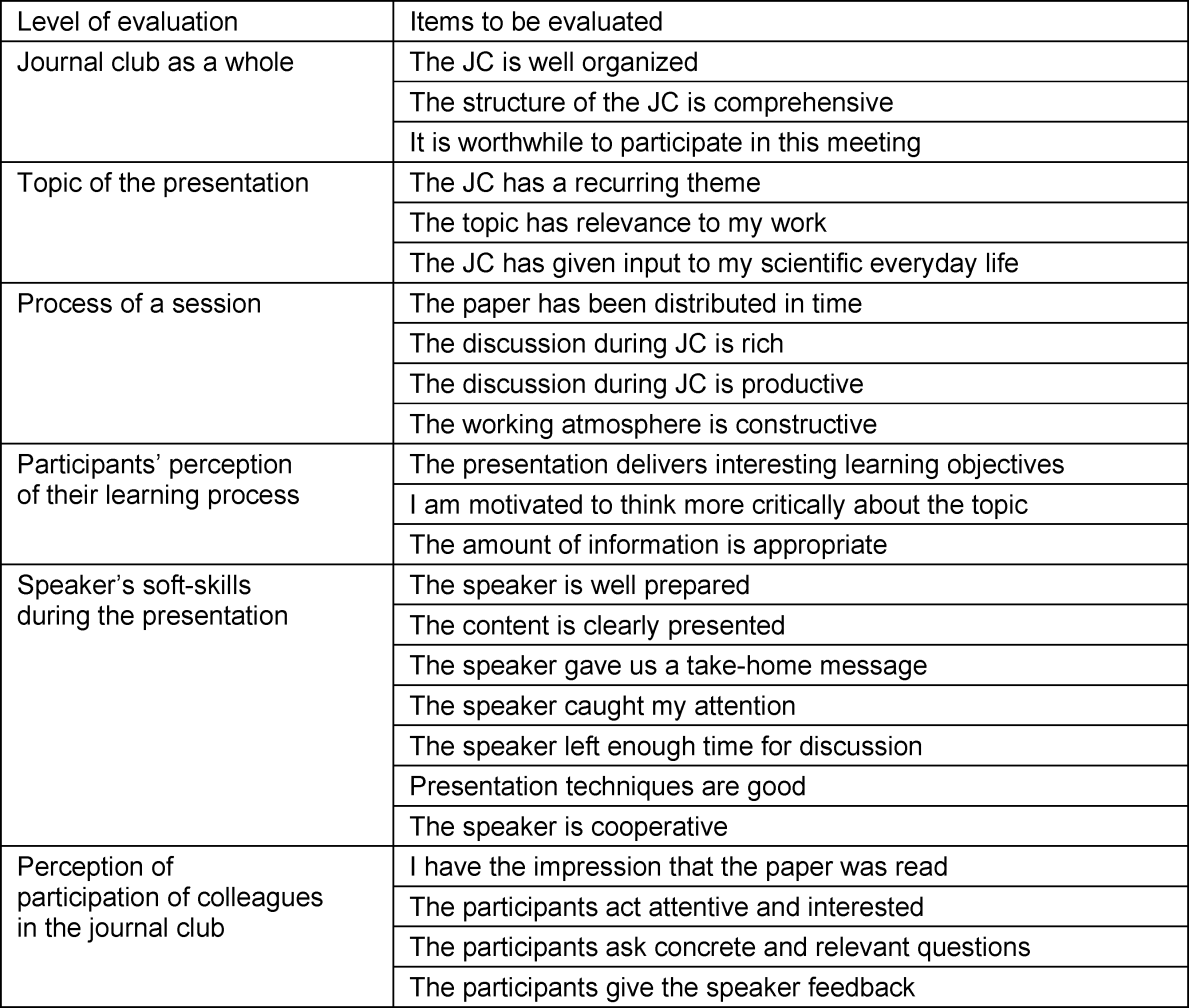
Questionnaire to evaluate perception of journal club sessions

**Table 2:**
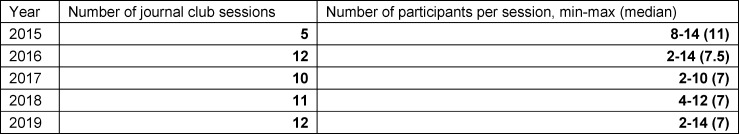
Number of evaluated journal club sessions and participants
